# On the nonlinearity of the foreperiod effect

**DOI:** 10.1038/s41598-024-53347-y

**Published:** 2024-02-02

**Authors:** Amirmahmoud Houshmand Chatroudi, Giovanna Mioni, Yuko Yotsumoto

**Affiliations:** 1 Department of Life Sciences, The University of Tokyo, Tokyo, Japan; 2 Department of General Psychology, University of Padua, Padua, Italy

**Keywords:** Perception, Sensory processing

## Abstract

One of the frequently employed tasks within the implicit timing paradigm is the foreperiod task. The foreperiod is the time interval spanning from the presentation of a warning signal to the appearance of a target stimulus, during which reaction time trajectory follows time uncertainty. While the typical approach in analyzing foreperiod effects is based on linear approximations, the uncertainty in the estimation of time, expressed by the Weber fraction, implies a nonlinear trend. In the present study, we analyzed the variable foreperiod reaction times from a relatively large sample (n = 109). We found that the linear regression on reaction times and log-transformed reaction times poorly fitted the foreperiod data. However, a nonlinear regression based on an exponential decay function with three distinctive parameters provided the best fit. We discussed the inferential hazards of a simplistic linear approach and demonstrated how a nonlinear formulation can create new opportunities for studies in implicit timing research, which were previously impossible.

## Introduction

Among the tools a researcher is equipped with to assess how time shapes perception and action, there exists the foreperiod task^1^. Foreperiod (FP) is the duration between a warning stimulus and a subsequent target stimulus. When the FP is kept constant within the blocks, the reaction time (RT) to the target stimulus increases as FP lengthens (fixed foreperiod effect), whereas a variable foreperiod shortens RT as FP lengthens (variable foreperiod effect^2–8^). Due to these temporal characteristics, the foreperiod task has been widely used in investigating the change of implicit timing mechanisms with aging (e.g. in children^9,10^, in healthy elderlies^11,12^, and in elderlies with cognitive decline^13^) or among clinical populations (e.g. schizophrenia^14^, Parkinson^15^, and autism^16^).

The increase of RT with duration in fixed foreperiod tasks has been collectively attributed to the clock-time uncertainty (scalar property of time^1,6,17,18^). However, there's an ongoing dispute regarding the mechanism driving the decrease in RT observed in the variable foreperiod task. This debate centers on two perspectives: one involves the conditional probability of target occurrence (hazard-based theories^19–24^), while the other focuses on the relative frequency of memory traces, as proposed by the Multiple Trace Theory of Temporal Preparation (MTP^17,25,26^). Nevertheless, in explaining the variable FP effect, both theories converge on considering the role played by time uncertainty.

In hazard-based studies, the hazard function (the probability of target occurring given it has not occurred yet over the unit of time^27,28^) is blurred according to the Weber ratio^22,29^. This temporal blurring accounts for the uncertainty associated with the estimation of elapsed time^30^, and thus transforms the objective hazard function into a subjective anticipation function^31^. The subjective hazard function is negatively correlated with the RT, and its neural representations in the cortex can be found^22,31^. Moreover, MTP theory^17^ and its precursor (trace conditioning^32^) have also recognized the uncertainty associated with the elapse of time and have similarly incorporated the Weber ratio in their model formulations.

**Figure 1:**
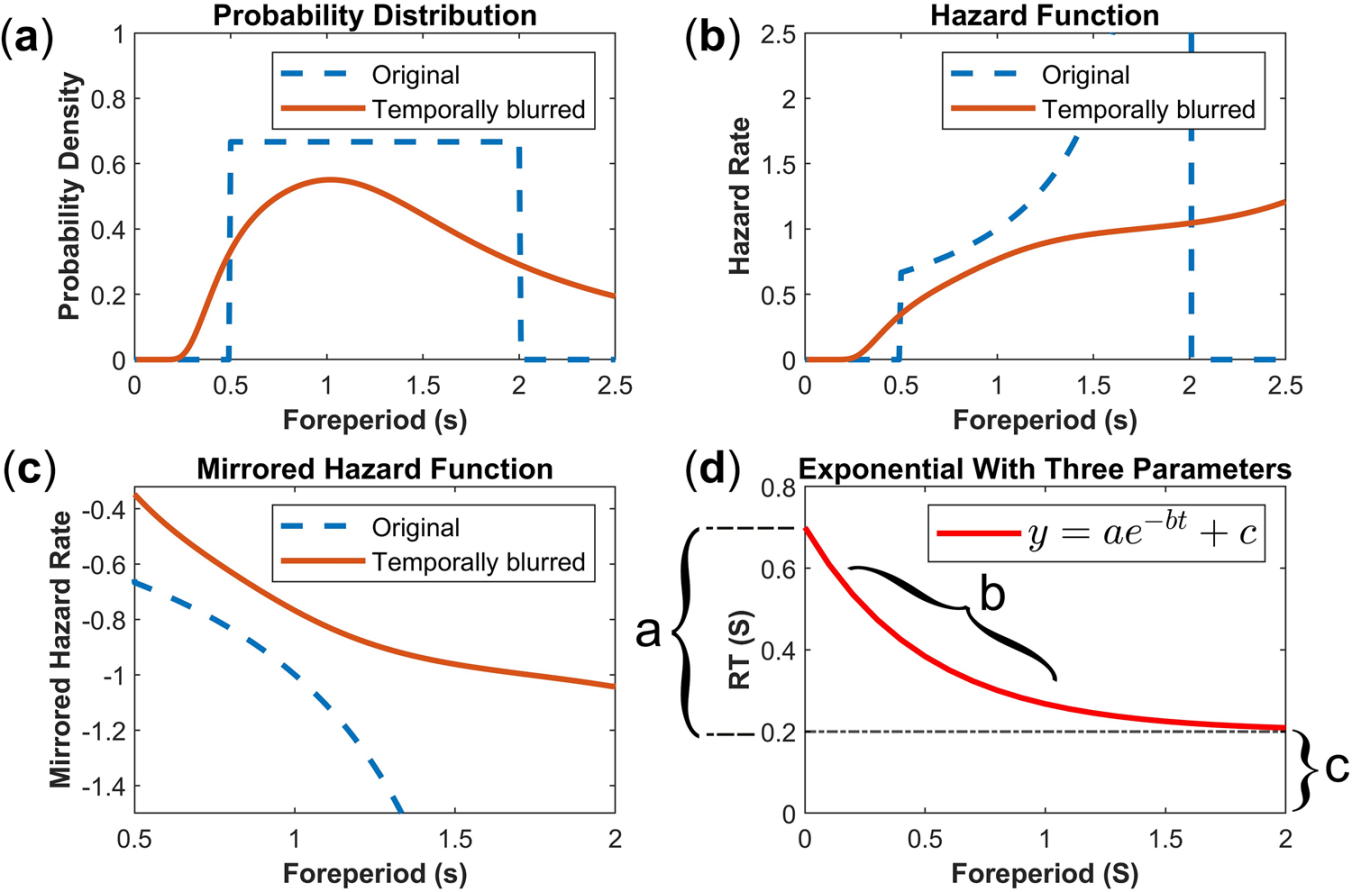
The blurring effect of applying Weber ratio to the objective hazard rate^22,35^. Panel (**a**) indicates a foreperiod range of 0.5 to 2 s, where the probability distribution of foreperiod intervals follows a uniform distribution. The blue dashed line represents the objective probability distribution. The red solid line shows the same distribution blurred according to the Weber ratio (φ = 0.49). Panel (**b**) shows the hazard function derived from panel (**a**). Note that the steep slope of blurred hazard rate in the beginning gradually decreases over longer durations. Panel (**c**) shows the inverted hazard function (mirrored; multiplied by − 1). The mirrored blurred hazard rate correlates positively with RT patterns in variable FP tasks. Note the steeper slope of mirrored blurred hazard rate in shorter durations. Panel (**d**) shows the potential nonlinear model for capturing the variable FP effect. Parameter ‘a’ in this exponential function is the scaling factor. This parameter corresponds to the amount participants can reduce their RT over the span of FP (the range of RT modulation). Parameter ‘b’ is the rate of decay, corresponding to the size of FP effect. Parameter ‘c’ is a constant (asymptote) corresponding to the motor/cognitive limitation. Thus, the assumption is that participants cannot improve their RT by reaching to zero, rather they will fixate at a constant RT due to motor/cognitive limitations. Note that the hazard function in panel (**b**) is derived from the continuous uniform probability density in panel (**a**). Therefore, the hazard function in panel (**b**) corresponds to the instantaneous rate of event occurrence given it has not occurred yet^28,36^. This value can exceed 1.

The Weber ratio dictates that the standard deviation of estimation linearly increases with the mean duration^18,30^. Therefore, within a given temporal interval, the passage of time across shorter durations is more discernible than the passage of time across longer durations^33,34^. In the context of the variable FP task (where FP durations are presented based on a uniform probability distribution shown in Fig. 1a), this means that the time passage across shorter durations increases the hazard rate (Fig. 1b) much faster than the passage of time across longer durations. Consequently, since reaction times are negatively correlated with the subjective hazard function, the relation between FP and RT will exhibit a negatively accelerating pattern^32,34^, as depicted in Fig. 1c, and potentially takes the form of an exponential decay function^17^ (illustrated in Fig. 1d). This is because shorter time intervals reduce the probability uncertainty faster, resulting in a quicker acceleration of RT (steeper slope in the first half of FP duration in Fig. 1c,d), while longer time intervals convey less information in resolving the probability uncertainty, leading to a slower acceleration of RT (gentler slope in the second half of FP duration in Fig. 1c,d).

The exponential decay pattern of FP effect is also implicated by the MTP theory^17^. The MTP theory assumes that the stored strength of activation for each moment in time (irrespective of their recency weight), is scaled based on the time uncertainty (the Weber ratio). That is, within the FP range, as the moment of target appearance (critical moment) becomes more remote from the onset of the warning stimulus, the peak strength of each moment’s activation becomes lower, and temporally more dispersed (forming a gamma distribution of activations^32,37^). Thus, the memory traces of shorter durations (i.e. earlier critical moments) which are formed from higher and more precise peaks of activations increase the preparatory state faster than the memory traces of longer durations (which result from lower peaks and more dispersed activations). This nonlinear increase in the preparatory state would then translate into the RT patterns in a form that resembles an exponential decay function (see Fig. 3c,e in Los et al.^17^). Hence, regardless of whether the FP effect arises from a subjective hazard function or the weighted strength of memory traces, the introduction of time uncertainty in these explanations assumes an inherent nonlinear pattern for the FP effect.

Despite early descriptive efforts in modeling the non-linearity of the FP effect^2,38–40^, the typical approach in analyzing the relation between RT and FP has stagnated at the assumption of linearity (applying analysis of variances (ANOVAs) and linear regressions to RTs or log-transformed RTs). In this formulation, the intercept differences between two conditions (or the main effect in an ANOVA) is regarded to reflect an additive change in the pattern of FP which is mainly attributable to a cognitive cost, motor limitation or a general slowing down of the RT^11,13,38,41,42^. The slope difference (or an interaction in an ANOVA) is then interpreted as a measure of change in the size of the FP effect between conditions^9,13^. However, applying a linear analysis to a phenomenon that is nonlinear by nature can pose serious statistical and inferential problems (see “Discussion”).

One inferential difficulty observed in variable FP studies is that between conditions and/or age groups, one population is generally slower, and consequently, has more room for reducing its RT over the duration of the foreperiod^11,13,33^. In such cases, the RT pattern results in interaction effects (or slope differences) which one may ascribe to the changes in the size of FP effect (i.e. changes in the amount of RT reduction per unit of time). However, this pattern can be alternatively explained by a simple increase in the amount of RT that can be reduced in a FP task (increase in the range of RT modulation, parameter ‘a’ in Fig. 1d), leaving the size of FP effect (parameter ‘b’ in Fig. 1d) unaltered. Therefore, it is conceivable that an interpretable model of the FP effect is required to have at least three parameters: a nonlinear parameter for quantifying the rate of RT decay over time (indicating the size of FP effect), a multiplicative parameter for capturing the range of RT modulation, and a constant that can capture the motor/cognitive limitation factors.

Hence, in the present study we attempted to shed light on whether linear approximations, widely used by the implicit timing and RT studies, can sufficiently capture the pattern of variable FP data. In doing so, we compared the fit of linear regression with different nonlinear regression models using Bayesian information criterion (BIC^43^) in a relatively large sample to find which model better describes the FP effect.

## Results

**Figure 2:**
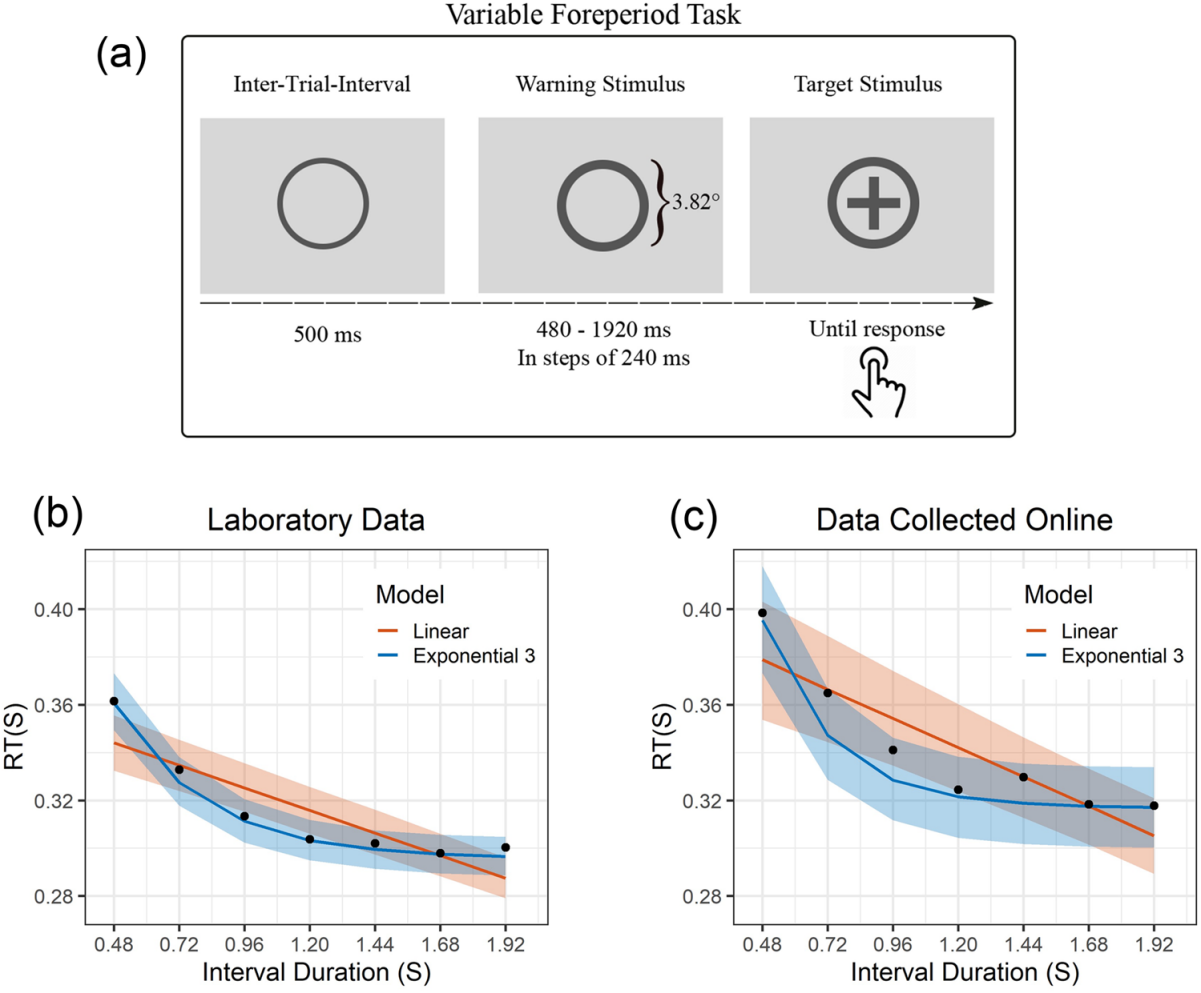
Task specifications (**a**) and group-level regression fits for data collected in the laboratory (**b**), and online (**c**). The shaded areas in (**a**,**b**) reflect 95% confidence bounds.

To assess whether the FP effect is better described by linear or nonlinear functions, reaction time data were collected from a variable foreperiod task (Fig. 2a) in the laboratory (n = 69) and online settings (n = 40). The reaction time data were subsequently fitted by mixed-effect linear and nonlinear regressions. Importantly, among the nonlinear formulations (see Method), the nonlinear equivalent of the commonly used linear analysis of log-transformed RT^9,13,16^ (corresponding to the Exponential 1 model) and log–log transformed RT (corresponding to the Power 1 model) were included (see Supplementary Materials for details). Moreover, among the various nonlinear formulations, we hypothesized that an exponential function with three parameters provides the best fit of RT data.

The model comparison results from the data collected in the laboratory (Table 1) and online settings (Table 2) both show that the linear model provided the worst fit relative to the best-fitting model based on BIC (BF01 = 5.15e46 and 2.55e14 for laboratory and online settings, respectively). The best-fitting model both for the data collected in the laboratory and online as hypothesized was the exponential function with three distinctive parameters (BF for Exponential 3 versus the second-best fitted model = 3.77e14 and 212.94 for laboratory and online datasets, respectively).

**Table 1:** Comparison of models fitted to the data collected in the laboratory (in-person).

Model	df	AIC	ΔAIC	AICc	BIC	ΔBIC	BIC weight	BF
Exponential 3	7	− 2454.48	0	− 2454.25	− 2425.22	0	1	1
Power 3	5	− 2378.99	75.49	− 2378.87	− 2358.09	67.13	0	3.77e14
Power 1	5	− 2372.92	81.56	− 2372.8	− 2352.02	73.2	0	7.85e15
Exponential 2	6	− 2353.41	101.08	− 2353.23	− 2328.33	96.9	0	1.10e21
Power 2	5	− 2348.71	105.78	− 2348.58	− 2327.81	97.42	0	1.42e21
Exponential 1	6	− 2249.71	204.77	− 2249.54	− 2224.63	200.59	0	3.61e43
Linear	6	− 2235.19	219.29	− 2235.01	− 2210.11	215.11	0	5.15e46

The rows are sorted according to BIC. The winning model based on BIC and AIC is Exponential 3. All digits shown are rounded to two decimal places. BF corresponds to the Bayes Factor in support of the Exponential 3.

**Table 2:** Comparison of models fitted to the data collected online.

Model	df	AIC	ΔAIC	AICc	BIC	ΔBIC	BIC weight	BF
Exponential 3	7	− 1161.71	0	− 1161.3	− 1136.27	0	0.99	1
Exponential 2	6	− 1147.35	14.36	− 1147.05	− 1125.54	10.72	0	212.94
Power 1	7	− 1149.66	12.05	− 1149.25	− 1124.21	12.05	0	413.97
Power 2	7	− 1137.74	23.97	− 1137.33	− 1112.3	23.97	0	160,411.3
Exponential 1	7	− 1109.33	52.38	− 1108.91	− 1083.88	52.38	0	2.37e11
Power 3	6	− 1104.68	57.03	− 1104.37	− 1082.87	53.4	0	3.94e11
Linear	7	− 1095.37	66.34	− 1094.96	− 1069.92	66.34	0	2.55e14

The rows are sorted according to BIC. The winning model based on BIC and AIC is the Exponential 3. All digits shown are rounded to two decimal places. BF corresponds to the Bayes Factor in support of the Exponential 3.

Moreover, results of model comparisons also indicated that the log transformation function (corresponding to the Exponential 1 model, see Supplementary Materials), substantially underfitted the data relative to the exponential with three parameters (BF01 = 3.61e43 and 2.37e11 for laboratory and online datasets, respectively). Similarly, log–log transformation function (corresponding to the Power 1 model, see Supplementary Materials) performed poorly relative to the Exponential 3 model (BF01 = 7.85e15 and 413.97 for laboratory and online datasets, respectively).

Figure 2 shows the group-level fit of Exponential 3 and linear functions for laboratory and online datasets. Subject-level fits of Exponential 3 to both datasets (Figs. S1 & S2), as well as estimated coefficients (Tables S1 & S2) can be found in the supplementary materials.

## Discussion

In the present study we aimed to evaluate whether the conventional linear regression, commonly used in FP research, can achieve comparable performance to nonlinear regression models when fitting the variable FP effect. Additionally, we aimed to unravel which nonlinear formulation of variable FP effect provides the best fit. For this purpose, data from a relatively large sample of participants in a controlled laboratory setting, and subsequently, in an online setting was collected and analyzed. Our hypothesis was that the best-fitting model would be an exponential function with three distinctive parameters: a nonlinear parameter for capturing the negatively accelerating decay rate of FP effect (parameter ‘b’ in Fig. 1d). This parameter corresponds to the size of FP effect. A multiplicative term for capturing the range of RT modulation (parameter ‘a’ in Fig. 1d). This parameter is the scaling factor. Finally, an additive constant term for capturing the motor/cognitive limitation factors (parameter ‘c’ in Fig. 1d). This parameter would be the asymptote, and thus captures the amount of RT that does not change with FP duration (Fig. 1d). Our results unequivocally demonstrated that both in the laboratory and online settings the linear model provided the worst fit of the variable FP effect, whereas an exponential model with three parameters outperformed all other models.

The pattern of an exponential decay function fits well with the nonlinearity imposed by the Weber ratio^22^: the steeper slope of RT improvement during shorter intervals versus the gentle slope of RT decrease during the longer intervals of a given FP. Relatedly, it has been shown that practice can substantially reduce the Weber ratio, changing the negatively accelerating function into a negative linear relation between RT and FP^35^. The negatively accelerating decay of FP effect also aligns well with the finding that larger range and smaller average FP increases the FP effect^33^. Moreover, the nonlinearity of FP effect has also been attributed to the more frequent subjective representation of medium durations^1^. Thus, it is proposed that such an unbalanced subjective distribution diminishes the RT differences between the medium and longer durations of an FP.

The early studies attempting to model the non-linearity of variable FP effect remained at a descriptive level. Niemi^38^ only tangentially illustrated that the variable FP effect can be captured by an exponential function with two parameters. Polzella et al.^40^ found that the FP effect decreased linearly in conditions with no catch trials while it followed a quadratic trend in conditions with catch trials. It is worth noting that these classic studies relied on collecting excessive RT data (more than 100 trials per condition and foreperiod) from a few participants (n = 4).

**Figure 3:**
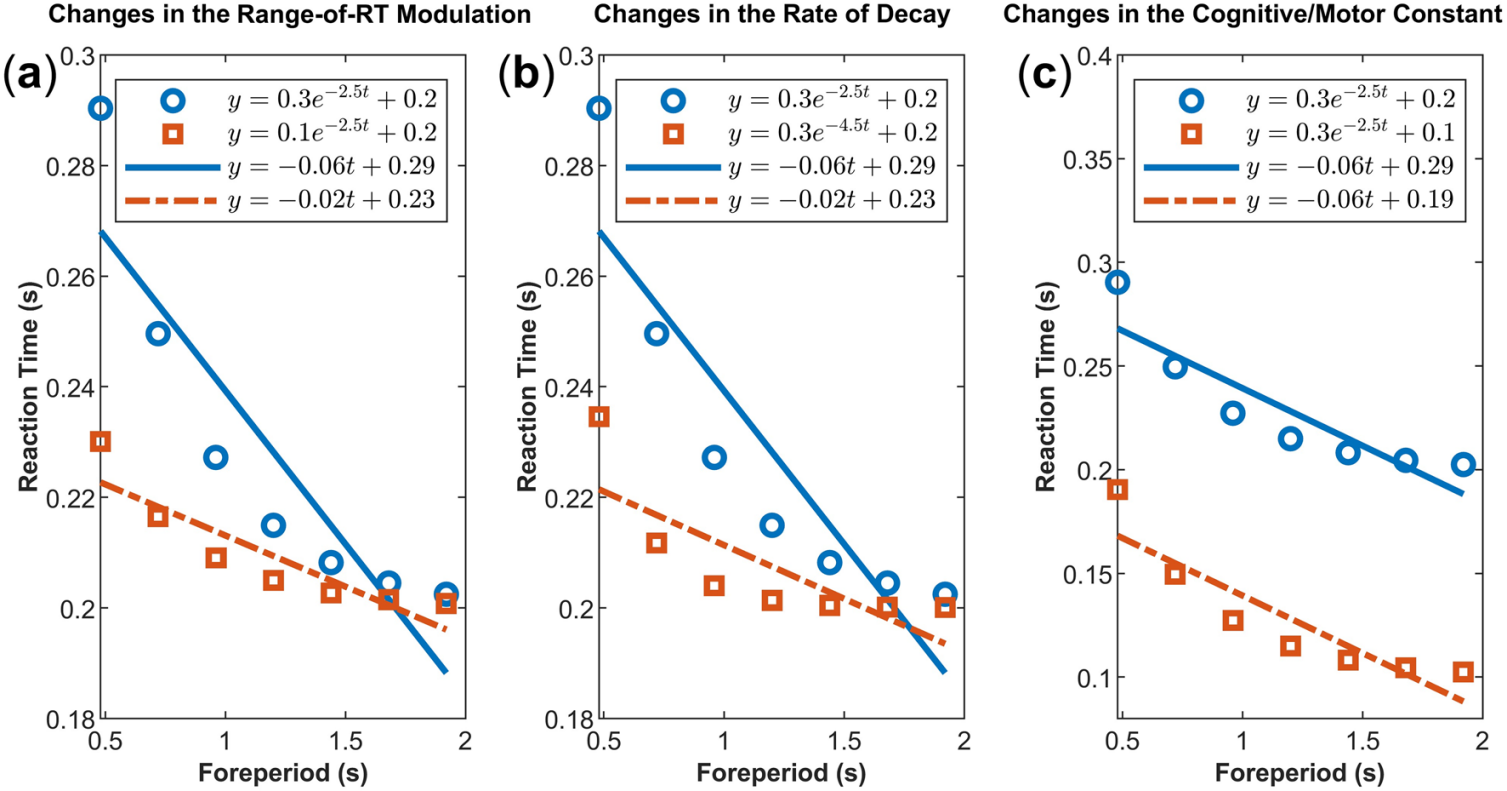
The interpretability problems of fitting a linear model to the variable FP effect. The blue circles are the RT points generated assuming an exponential function with three parameters. The lines are the best linear fits. In panel (**a**), the red squares show a change in the scaling factor parameter of the exponential function (i.e. the range of RT modulation). The red dashed line represents the best linear fit. Compared to the best fit of the original data (the blue solid line), both the intercept and slope of the red dashed line have simultaneously changed. In panel (**b**), the red squares represent a change in the rate-of-decay of the original data (blue circles). Note that the slope and intercept of the linear fit (red dashed line) are both affected (compared to the blue solid line). In panel (**c**), the red squares indicate a change in the constant (asymptote) of the original exponential function. It is only in this scenario that the intercept of a linear function selectively captures the change without affecting the slope (generally representing a motor/cognitive cost, see text). Moreover, comparison of the red dashed lines between panel (**a**) and panel (**b**) illuminates that a linear model can remain insensitive to selective changes in the parameters of an underlying nonlinear function (in this case, scaling factor and rate-of-decay of an exponential function resulted in the same linear fits, i.e. red dashed lines with equal equations).

Applying linear analysis to a naturally nonlinear phenomenon can lead to significant statistical and inferential challenges. This is because a change in a parameter of an underlying nonlinear phenomenon (as might be the intention of an experimental manipulation) can affect the slope and intercept of a fitted linear regression simultaneously. This, in turn, confounds the interpretation of the linear parameters and smears the true underlying effect. Figure 3 demonstrates that a selective change in the scaling factor (panel a) or the rate of decay (panel b) of an underlying exponential function (red squares compared to blue circles) can simultaneously affect the slope and the intercept of the best-fitting linear regression equations (red dashed lines compared to blue solid lines). Moreover, a true difference between conditions might go unnoticed as identical linear equations can accommodate changes from different nonlinear parameters (in panels a and b, the best-fitting linear red dashed lines have identical equations even though the datapoints (i.e., red squares in each panel) were created by selectively changing the scaling factor or the rate of decay of the underlying exponential function (i.e. blue circles), respectively).

To overcome the nonlinearity of RT data in foreperiod tasks some methods have been proposed^42,44,45^. Among them, the log transformation of RT is a common approach^9,13,16^. However, our results clearly demonstrated that log (Exponential 1) and log–log (Power 1) transformations substantially underfit the data relative to the exponential function with three parameters. Therefore, these makeshift methods, in addition to inferential difficulties, are suboptimal solutions to the nonlinearity of variable FP effect.

**Figure 4:**
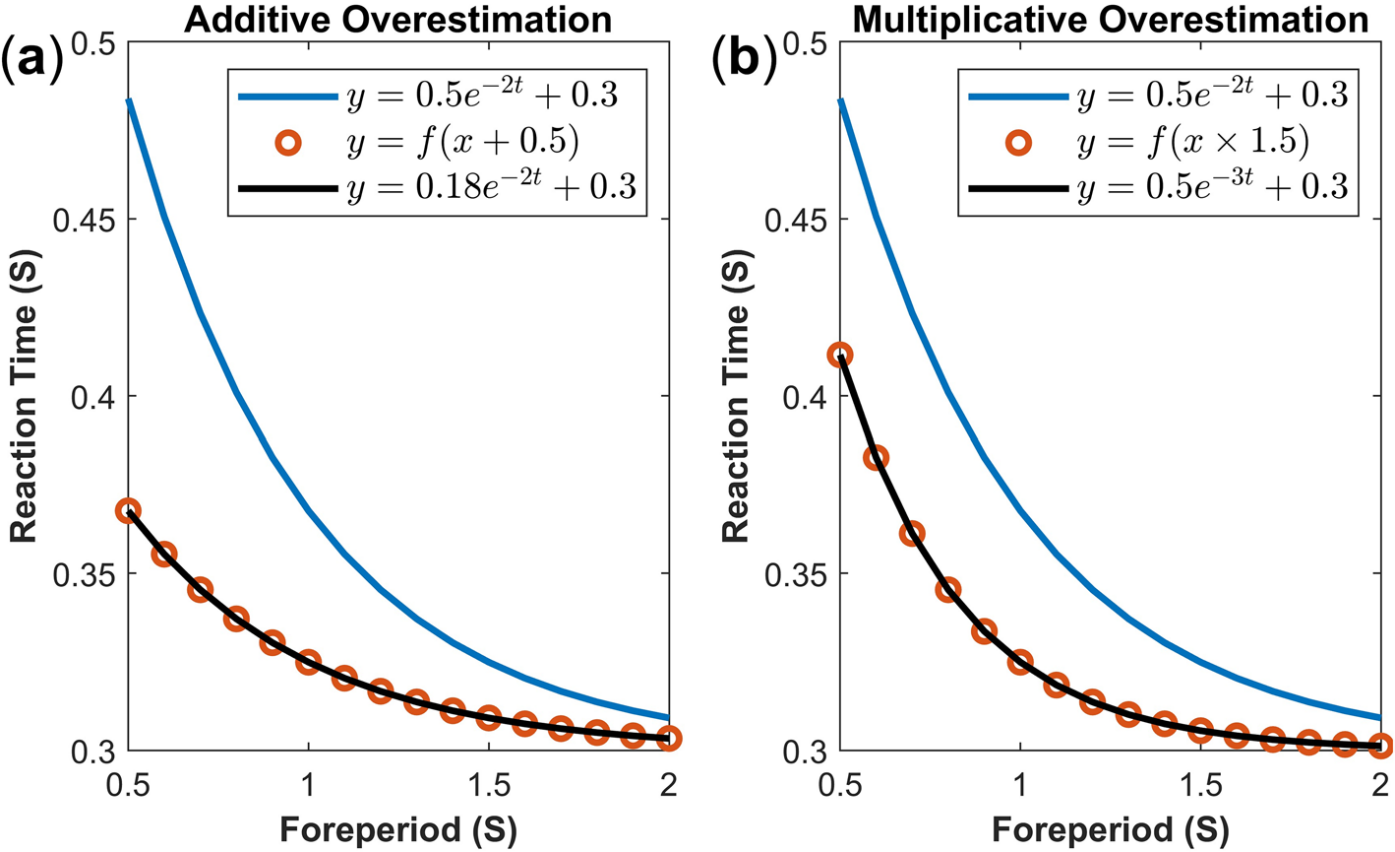
An exponential function with three parameters can selectively capture additive and multiplicative (under-) overestimation of time in a within-subject design. The blue line reflects an arbitrary reference function. The red circles represent RT under additive or multiplicative overestimations. The black line reflects the fit of the Exponential 3 function. Under the additive overestimation (panel (**a**)), foreperiod intervals are anticipated earlier than they should by a constant value (e.g. by 0.5 s as reflected by $y=f(x+0.5)$; red circles relative to the blue reference line). Such an overestimation is selectively captured by changes in the scaling factor parameter (scaling factor parameter of the black line relative to the blue reference function). Under the multiplicative overestimation (panel (**b**)), foreperiod intervals are expected earlier than they should by a constant rate (e.g. by 50% as reflected by $y=f(x\times 1.5)$; red circles relative to the blue reference line). Such an overestimation is independently captured by the rate-of-decay parameter of the Exponential 3 function (black line relative to the blue reference function).

Fitting mixed-effect nonlinear regressions to FP effect can open new avenues for time perception research. Future studies can elucidate how each of the three parameters proposed here would vary with age, different attentional capacities, task difficulties, and task modalities (for a review, see Niemi & Näätänen^1^). More importantly, it is vital to determine whether the non-linearity pattern of FP is held constant among age groups and task conditions, or it varies with different conditions (for example, a power function may better fit FP data from elderlies due to differences in their time uncertainties). Lastly, in a within-subject design, the proposed exponential function possesses the power to capture over- and underestimations of FPs among conditions. This was not so far attainable due to the crudity of the linearity assumption. As depicted in Fig. 4, in a *within-subject design*, if one condition leads to the overestimation of time, the rate-of-decay parameter can selectively capture the multiplicative overestimation (changes in the rate of the clock^46,47^) while the scaling factor parameter captures the additive overestimation (changes in the switch latency^47^). These overestimations are captured independently by each of the aforementioned parameters without affecting the motor/cognitive constant. Hence, nonlinear regression models create new opportunities in implicit timing research, contributing to a better understanding of how characteristics of ‘time’ affect the pattern of FP effect.

### Limitations

The present study aimed to clarify the nonlinear shape of the variable foreperiod effect based on the RTs in the current trials. However, it has been suggested that foreperiod intervals in the previous trials also contribute to the downward trend of FP-RT function in the current trials^48^. This contribution has been attributed to the foreperiod’s asymmetric sequential effect^17,32,34,48^. It is noteworthy that the shape of such an asymmetric sequential effect also exhibits a nonlinearity pattern that is qualitatively similar to an exponential function with three parameters (e.g. Fig. 5 in Los et al.^32^). However, whether the nonlinear pattern of the asymmetric sequential FP effect (on top of temporal anticipation) directly contributes to the exponential shape of FP-RT remains unclear. In the present study, the trial sequence was not organized in a way that we can specifically address this hypothesis. Therefore, it remains the topic of future investigations to qualify the nonlinear shape and contributions of sequential effects to the FP-RT function in current trials.

A corollary of studies investigating the sequential effects has been that the variable foreperiod effect is not solely determined by temporal preparation. Rather, it is a context-dependent effect relying on different variables. For instance, previous research has shown that a shift in the modality of the warning stimuli, as well as, event-specific (sequential action) biases^49^ can change the slope of the FP-RT effect in current trials^50^. More importantly, the temporal context (whether the previous trials have consistently been short or long) can modulate the slope of the FP-RT effect in a manner that is compatible with an arousal-based explanation^51^. In the present study, we did not investigate the contributions of context-dependent variables. Nonetheless, the approach in quantifying the context (sequential) variables affecting the FP-RT has been based on linear analyses^17,32,34,48–51^. Thus, it is worthwhile to assess whether the nonlinear pattern of the FP-RT effect is qualitatively sensitive to context variables (whether under the manipulation of different context variables, distinct nonlinear functions describe the FP effect). Moreover, should the exponential function with three parameters provide the best fit, it is important to discern which parameters are selectively impacted. This is because, as pointed out before (Fig. 3a), a change in the slope of the FP-RT function under the linear analysis (given the underlying function is exponential) might not be due to a true change in the foreperiod effect.

In the present study, FP durations (less than two seconds in duration) were investigated using a simple RT task. Thus, whether the findings of the present study can be generalized to FP-RT patterns in choice RT tasks, and/or other temporal ranges requires further investigations. Specifically, it is probable that under larger FP ranges the assumption of the asymptotic decline of RT (parameter ‘c’ in Fig. 1d) might not hold (e.g. due to fatigue). This point might especially be valid since under foreperiod ranges in the order of minutes, an FP task bears similarities to the Psychomotor Vigilance Task (PVT^52^) where goal-setting aspects play a vital role^53^.

## Method

### Participants

Hundred ten university students participated. Seventy participants were tested in person at the Department of General Psychology (University of Padova, Italy); data from one subject was removed from further analysis (see analysis), leaving 69 participants in the final analyses (mean age 24 years old SD = 2.37; male = 34; mean years of education = 14.92; SD = 1.71). The remaining 40 participants (mean age 22.90 years old SD = 2.45; male = 11; mean years of education = 14.30; SD = 1.91) performed the FP task remotely (online).

### Apparatus

The stimuli consisted of a grey circle (diameter = 3.82°) and a grey cross (3°), displayed at the center of a lighter grey background screen. A thin circle was initially displayed for 500 ms (Inter-Trial-Interval, ITI), followed by a thicker circle that could last for one of the following interval durations: 480, 720, 960, 1200, 1440, 1680, or 1920 ms. After the duration had elapsed, a cross appeared in the center of the circle for 500 ms. Participants were instructed to press the spacebar as fast as possible whenever the cross appeared in the center of the thicker circle, following the protocol commonly used in variable foreperiod tasks. Participants were not provided with any information about stimulus durations.

### Procedure

Participants tested in-person were seated in a quiet room at an approximate distance of 60 cm from the computer screen (15.6″) that produced and recorded experimental events via PsychoPy Software^54^. For those tested online, the recruitment process was carried out either in class or using the common procedure adopted to recruit participants at the Department of General Psychology, University of Padova, Italy. Upon agreeing to participate in the study, these individuals received a link to perform the task. The experiment, created using PsychoPy^54^, was identical to the in person testing and was hosted by Pavlovia (Open Science Tools, Nottingham, UK). Participants tested remotely were instructed to perform the task in a quiet room and to be seated approximately 60 cm from their computer screen.

All participants had normal or corrected-to-normal vision and normal hearing. Participation was voluntary, and no compensation was provided for participating in the study. All participants signed an informed consent prior to the participation, in accordance with the Declaration of Helsinki. The experiment was approved by the Ethics Committee of the Department of General Psychology of the University of Padova.

Our experimental protocol consisted of four blocks of 42 trials each (with six repetitions for each temporal interval). Thus, the number of trials per foreperiod was set to 24 repetitions, which is in general agreement with the number of trials used per foreperiod in previous studies of simple RT task^7,11,13,15,16,19,20,55^. In the online experiment, four blocks were administrated for each subject as planned. For the data collected in the laboratory, the majority of participants underwent four blocks (n = 36 out of 70 participants). However, due to a technical issue, 25 (out of 70 participants) went through five blocks and the remaining 9 (out of 70 participants) underwent three blocks. All participants, irrespective of the number of blocks they underwent, were included in the analysis reported here (for a detailed analysis of each subgroup, see Supplementary Materials). To acquaint participants with the task, a practice phase was included, consisting of 7 trials, with one presentation for each temporal interval.

### Analysis

Prior to fitting regression models, for each participant and for each FP, we collapsed all trials. Next, trials with extreme outliers (three times the interquartile interval below the first quartile (Q1) or above the third quartile (Q3)) were identified and removed^56^. This resulted in removal of 2.95% and 2.94% trials from the laboratory (in-person) and online datasets, respectively. The RTs in the remaining trials, per participant and per FP, were averaged and used for subsequent analyses.

The focus of the present study was to address the neglected nonlinearity in the mainstream average-based approaches to the FP-RT analysis. Therefore, in the present study, we summarized the data per participant and foreperiod by taking the average of RT distributions (following the standard analysis method of the mainstream previous research on FP effect^8,9,19,32,34^). However, using distributional analyses (e.g. drift–diffusion models^57^, or event history analysis^58^), might provide additional insights into the underlying nonlinearity of summarized RT distributions.

After averaging trails per participant and per FP, outlier participants were detected as follows: first, a linear regression was separately fitted for each participant; subsequently, participants whose fitted slope showed an extreme upward trend (three times the interquartile interval below Q1 or above Q3) were removed from further analysis. This procedure resulted in the removal of data from one participant in the laboratory dataset.

In order to compare the fit of the linear model against nonlinear formulations, a mixed-effect linear regression and several nonlinear mixed-effect regression models were created. The fitting procedure was based on the maximum likelihood method using nlme package (v3.1–153^59^ in R). The fixed effect in each model was always the intercept for each of the terms specified in the model’s formula (Table 3). Participants were specified as random effects, and the random structure was kept maximal whenever possible. The goodness-of-fit of maximal random structure was compared against the minimal structures and the assumption of independence of random effects was tested. Consequently, the model with the lowest BIC was selected. Heteroskedasticity was observed for most fits of data collected online. To overcome this, the variance function of all models was structured with Varpower function in nlme package^59^. For comparison between models, Akaike information criterion (AIC^60^), small sample Akaike information criterion (AICc^61^), BIC and BIC weights were reported. AIC is generally regarded as a liberal criterion, whereas BIC is a more conservative criterion that penalizes overfitting^59^. AICc is a corrected version of AIC that is proposed for small sample sizes. AIC and AICs values converge as sample size becomes larger^61^. ΔAIC is calculated by subtracting the AIC value for each model from the model with the minimum AIC^62^. Therefore, the best-fitting model, based on AIC, is forced to have zero ΔAIC. Similarly, ΔBIC is calculated by subtracting the BIC value for each model from the model with the minimum BIC (the best-fitting model). Therefore, the best model based on BIC is forced to have zero ΔBIC. ΔBIC values are then used to approximate the Bayes factor (BF) in support of the best-fitting model relative to other models^63^ (see Supplementary Materials). BIC weight is the relative likelihood of the model given the data (normalized to sum to 1).

**Table 3:** Model names and model equations used in the regression analysis.

Model name	Model equation
Linear	$Y=aX+c$
Power 1	$Y=a{X}^{-b}$
Power 2	$Y={X}^{-b}+c$
Power 3	$Y=a{X}^{-b}+c$
Exponential 1	$Y= a{e}^{-bX}$
Exponential 2	$Y={e}^{-bX}+c$
Exponential 3	$Y=a{e}^{-bX}+c$

In all models presented below, ‘Y’ is RT and ‘X’ is FP. Moreover, parameter ‘a’ reflects the range of RT modulation, parameter ‘b’ reflects the size of FP effect (RT reduction). Parameter ‘c’ reflects the motor/cognitive limitation constant. The models tested were as follows:

The nonlinear Power 1 model corresponds to fitting a linear regression to the log–log transformed RT (see Supplementary Materials). The nonlinear Exponential 1 model corresponds to fitting a linear regression to the log-transformed RT (see Supplementary Materials).

## Supplementary Information

Supplementary Information.

## Data Availability

Reported data and analysis scripts from all experiments are openly available on the Open Science Framework (https://osf.io/km27b/).
