# Author Correction: On the nonlinearity of the foreperiod effect

**DOI:** 10.1038/s41598-025-94097-9

**Published:** 2025-03-20

**Authors:** Amirmahmoud Houshmand Chatroudi, Giovanna Mioni, Yuko Yotsumoto

**Affiliations:** 1 Department of Life Sciences, The University of Tokyo, Tokyo, Japan; 2 Department of General Psychology, University of Padua, Padua, Italy

Correction to: *Scientific Reports* 10.1038/s41598-024-53347-y, published online 02 February 2024

The original version of this Article contained a repeated error, where the parameter ‘*a*’ was referred to as the y-intercept but this is now corrected to read the “scaling factor.”

The original article has been corrected.

