# Supplementary material for: On the nonlinearity of the foreperiod effect
Source: Sci Rep. 2024 Feb 2;14:2780. doi: 10.1038/s41598-024-53347-y (PMC10837441; doi:10.1038/s41598-024-53347-y)
Supplement: Supplementary file 1 — Supplementary Information. [file 41598_2024_53347_MOESM1_ESM.docx]

**Equation 1**. Fitting a linear regression to log-transformed RT equals to fitting the Exponential 1 model used in this study according to the following equation:

$$\log Y=bX+c$$

$$e^{\log Y}=e^{bX+c}$$

$$Y= e^{bX}\times e^{c}$$

simplifying $e^{c}$ to $a$,

$$Y={ae}^{bX}$$

**Equation 2**. Fitting a linear regression to log-log transformed RT equals to fitting the Power 1 model used in this study according to the following equation:

$$\log Y=b\log X+c$$

$$e^{\log Y}=e^{\log X^{b}+c}$$

$$Y= X^{b}\times e^{c}$$

Simplifying $e^{c}$ to $a$,

$$Y= {aX}^{b}$$

**Equation 3**. Bayes factors were derived from BIC according to the following equation:

$$BF01=exp({\Delta BIC}_{10}/2)$$

**
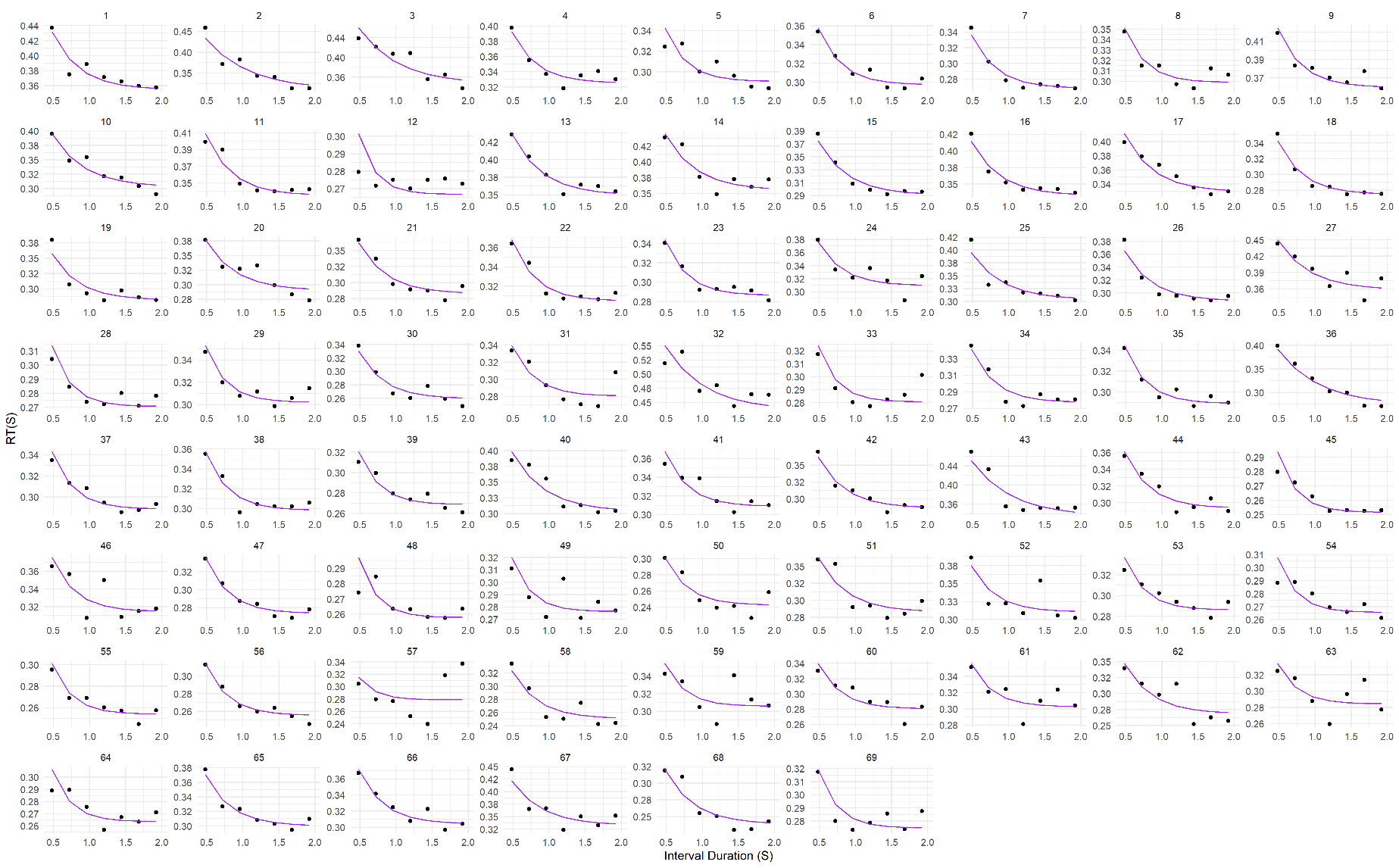
Figure S1**. The subject-level fit of laboratory data with Exponential 3.


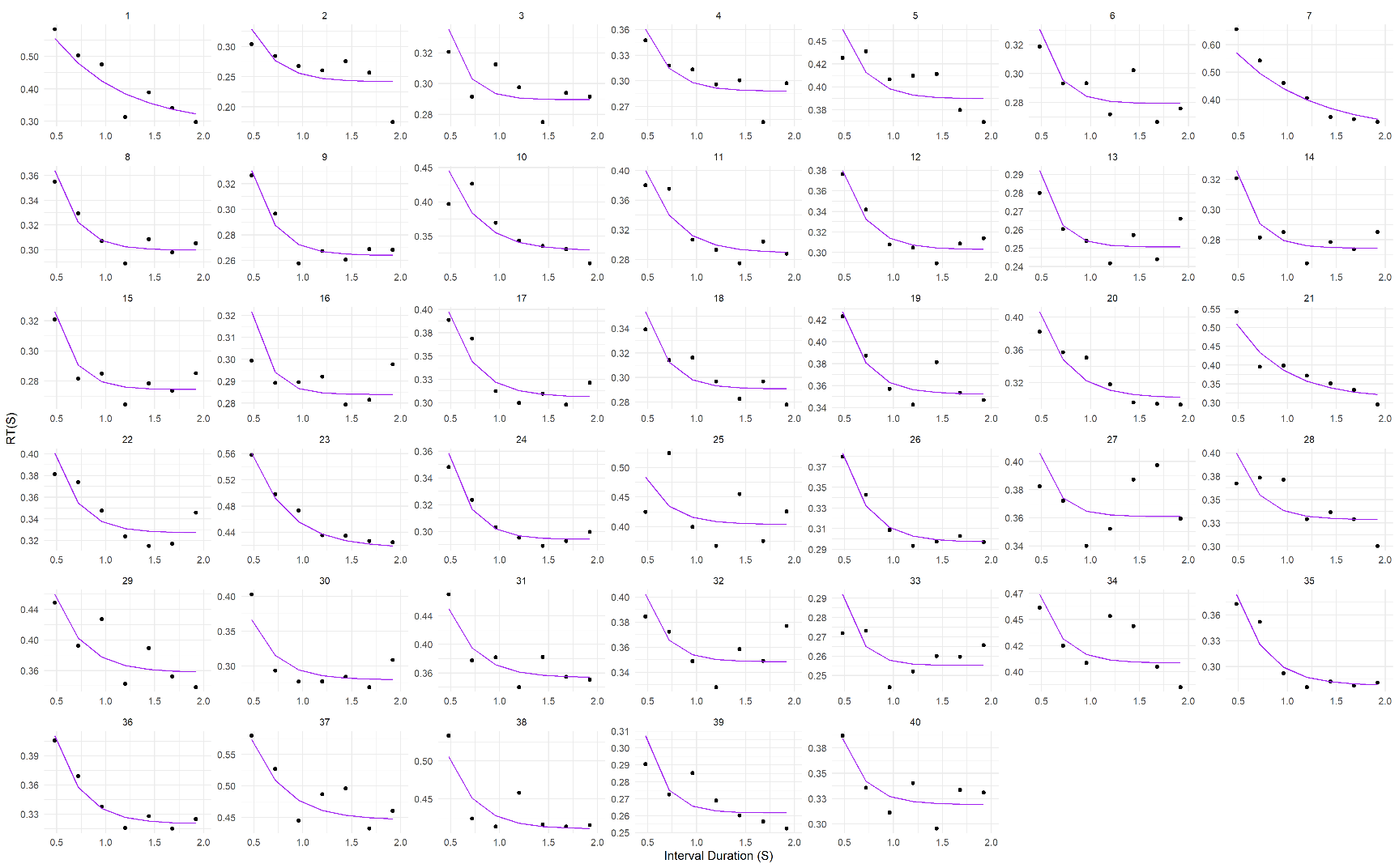


**Figure S2**. The subject-level fit of online data with Exponential 3.

**Table S1**. The estimated fixed-effect coefficients of Exponential 3 for laboratory data ($Y=ae^{-bX}+c$).

| Parameter | df | Value | Standard Error | P-value |
| --- | --- | --- | --- | --- |
| a | 412 | 0.2732 | 0.02158 | <0.001 |
| b | 412 | 2.9786 | 0.1956 | <0.001 |
| c | 412 | 0.29562 | 0.0043 | <0.001 |

**Table S2**. The estimated fixed-effect coefficients of Exponential 3 for online data ($Y=ae^{-bX}+c$).

| Parameter | df | Value | Standard Error | P-value |
| --- | --- | --- | --- | --- |
| a | 238 | 0.5240 | 0.0707 | <0.001 |
| b | 238 | 3.9438 | 0.3382 | <0.001 |
| c | 238 | 0.3162 | 0.0085 | <0.001 |

**Control Analysis**

The participants whose data were collected in the laboratory setting went through different number of blocks. While the majority of participants underwent the four-block regime (35 out of 69 participants), due to a technical issue, 25 (out of 69) participants underwent five blocks. Thus, the total number of trials for each of these participants was 210. Moreover, a subset of 9 (out of 69) participants went through three blocks, making the total of trials for these participants 126 (Table S4).

To ensure that the disparity in the number of blocks in the laboratory setting did not have a substantial effect on the results of model comparisons reported in the main text (Table 1), we conducted a control analysis. For this purpose, we re-ran the same analysis (reported in the main text) for each of the subset of participants that had the similar number of blocks (three groups).

The results are summarized in Table S4. Thus, in all subgroups of participants, the exponential 3 function was the best fit. The Bayes factor in support of the Exponential 3 relative to the second-best fitting function scaled with the number of participants in each subgroup. That is, the Bayes factor was the highest for the participants who underwent four blocks (n = 35; BF = 3.08e+07). Next, it was highest for the participants with five blocks (n = 25; BF = 1071.57). Lastly, for the participants with three blocks (n = 9), the support in favor of the Exponential 3 function was anecdotal relative to the second-best fitting function (BF = 1.58). More importantly, in all three subgroups, the linear function provided the worst fit. Similarly, Exponential 1 and Power 1, which correspond to the log and log-log transformations, respectively, underfitted the data. The detailed model comparison results are provided in tables S5, S6, and S7, respectively for each of the ‘four blocks’, ‘five blocks’, and ‘three blocks’ subgroups of participants.

**Table S3**. A summary of number of participants, blocks, and trials reported in the main text for laboratory and online experiments. BF corresponds to the Bayes factor in support of Exponential 3 relative to the second best-fitting model.

| Data | Number of participants | Number of Blocks | Total Number of Trial | BF in favor of EXP 3 |
| --- | --- | --- | --- | --- |
| **Online** | 40 | 4 | 168 | 212.94 |
| **Laboratory** | 70(69) | Between 3 to 5 | Between 126 to 210 | 3.77e14 |

**Table S4**. The detailed number of blocks, and trials which participants underwent in the laboratory experiment. BF corresponds to the Bayes factor in support of Exponential 3 relative to the second best-fitting model.

| Data | Number of participants | Number of Blocks | Total Number of Trial | BF in favor of EXP 3 |
| --- | --- | --- | --- | --- |
| Laboratory | 36 (35) | 4 | 168 | 3.08e+07 |
|  | 25 | 5 | 210 | 1071.57 |
|  | 9 | 3 | 126 | 1.58 |

**Table S5**. The comparison of models fitted to the subset of participants who underwent ‘four blocks’ in the laboratory experiment (n = 35 out of 69). The rows are sorted according to BIC. The winning model based on BIC and AIC is Exponential 3. All digits shown are rounded to two decimal places. BF corresponds to the Bayes Factor in support of the Exponential 3.

| Model | df | AIC | ΔAIC | AICc | BIC | ΔBIC | BIC weight | BF |
| --- | --- | --- | --- | --- | --- | --- | --- | --- |
| Exponential 3 | 8 | -1232.33 | 0 | -1231.72 | -1204.32 | 0 | 1 | 1 |
| Power 3 | 6 | -1190.84 | 41.49 | -1190.49 | -1169.83 | 34.49 | 0 | 3.08E+07 |
| Power 1 | 6 | -1183.31 | 49.02 | -1182.96 | -1162.3 | 42.01 | 0 | 1.33E+09 |
| Exponential 2 | 6 | -1175.87 | 56.46 | -1175.52 | -1154.86 | 49.46 | 0 | 5.48E+10 |
| Power 2 | 7 | -1178.46 | 53.86 | -1177.99 | -1153.96 | 50.36 | 0 | 8.63E+10 |
| Exponential 1 | 6 | -1129.58 | 102.75 | -1129.23 | -1108.57 | 95.74 | 0 | 6.17E+20 |
| Linear | 7 | -1126.38 | 105.94 | -1125.91 | -1101.87 | 102.44 | 0 | 1.76E+22 |

**Table S6**. The comparison of models fitted to the subset of participants who underwent ‘five blocks’ in the laboratory experiment (n = 25 out of 69). The rows are sorted according to BIC. The winning model based on BIC and AIC is Exponential 3. All digits shown are rounded to two decimal places. BF corresponds to the Bayes Factor in support of the Exponential 3.

| Model | df | AIC | ΔAIC | AICc | BIC | ΔBIC | BIC weight | BF |
| --- | --- | --- | --- | --- | --- | --- | --- | --- |
| Exponential 3 | 7 | -931.5 | 0 | -930.83 | -909.34 | 0 | 1 | 1 |
| Power 3 | 10 | -927.04 | 4.46 | -925.7 | -895.39 | 13.95 | 0 | 1071.57 |
| Power 1 | 5 | -897.52 | 33.98 | -897.16 | -881.69 | 27.65 | 0 | 1010800 |
| Exponential 2 | 6 | -892.3 | 39.2 | -891.8 | -873.31 | 36.04 | 0 | 6.68E+07 |
| Power 2 | 5 | -879.31 | 52.19 | -878.95 | -863.49 | 45.86 | 0 | 9.08E+09 |
| Exponential 1 | 5 | -838.14 | 93.36 | -837.78 | -822.31 | 87.03 | 0 | 7.91E+18 |
| Linear | 6 | -832.26 | 99.24 | -831.76 | -813.27 | 96.08 | 0 | 7.29E+20 |

**Table S7**. The comparison of models fitted to the subset of participants who underwent ‘three blocks’ in the laboratory experiment (n = 9 out of 69). The rows are sorted according to BIC. The winning model based on BIC and AIC is Exponential 3. All digits shown are rounded to two decimal places. BF corresponds to the Bayes Factor in support of the Exponential 3.

| Model | df | AIC | ΔAIC | AICc | BIC | ΔBIC | BIC weight | BF |
| --- | --- | --- | --- | --- | --- | --- | --- | --- |
| Exponential 3 | 5 | -303.19 | 0 | -302.14 | -292.47 | 0 | 0.56 | 1 |
| Power 3 | 5 | -302.27 | 0.92 | -301.22 | -291.56 | 0.92 | 0.35 | 1.58 |
| Power 1 | 5 | -298.52 | 4.66 | -297.47 | -287.81 | 4.66 | 0.05 | 10.3 |
| Exponential 2 | 5 | -296.54 | 6.65 | -295.49 | -285.83 | 6.65 | 0.02 | 27.76 |
| Power 2 | 5 | -295.05 | 8.14 | -294 | -284.34 | 8.14 | 0.01 | 58.46 |
| Exponential 1 | 5 | -282.95 | 20.24 | -281.9 | -272.23 | 20.24 | 0 | 24852.81 |
| Linear | 5 | -280.93 | 22.26 | -279.87 | -270.21 | 22.26 | 0 | 68270.04 |

Thus, all the results obtained from the whole sample that were reported in the main text were also present within each subgroup of participants with different blocks. Moreover, the strength of evidence in support of the results scaled with the number of participants present in each subgroup.
